# Impact of Fecal Microbiota Transplantation on Gut Bacterial Bile Acid Metabolism in Humans

**DOI:** 10.3390/nu14245200

**Published:** 2022-12-07

**Authors:** Jessica-Miranda Bustamante, Tyson Dawson, Caitlin Loeffler, Zara Marfori, Julian R. Marchesi, Benjamin H. Mullish, Christopher C. Thompson, Keith A. Crandall, Ali Rahnavard, Jessica R. Allegretti, Bethany P. Cummings

**Affiliations:** 1 Department of Surgery, School of Medicine, Center for Alimentary and Metabolic Science, University of California, Sacramento, CA 95817, USA; 2 Computational Biology Institute, Department of Biostatistics and Bioinformatics, Milken Institute School of Public Health, The George Washington University, Washington, DC 20052, USA; 3 Division of Digestive Diseases, Department of Metabolism, Digestion and Reproduction, Faculty of Medicine, St. Mary’s Hospital Campus, Imperial College London, London W2 1NY, UK; 4 Division of Gastroenterology, Hepatology and Endoscopy, Brigham and Women’s Hospital, Harvard Medical School, 75 Francis Street, Boston, MA 02115, USA; 5 Department of Biostatistics and Bioinformatics, Milken Institute School of Public Health, The George Washington University, Washington, DC 20052, USA

**Keywords:** bile salt hydrolase (BSH), bile acids, gut microbiota, metagenomics, fecal microbiome transplant (FMT)

## Abstract

Fecal microbiota transplantation (FMT) is a promising therapeutic modality for the treatment and prevention of metabolic disease. We previously conducted a double-blind, randomized, placebo-controlled pilot trial of FMT in obese metabolically healthy patients in which we found that FMT enhanced gut bacterial bile acid metabolism and delayed the development of impaired glucose tolerance relative to the placebo control group. Therefore, we conducted a secondary analysis of fecal samples collected from these patients to assess the potential gut microbial species contributing to the effect of FMT to improve metabolic health and increase gut bacterial bile acid metabolism. Fecal samples collected at baseline and after 4 weeks of FMT or placebo treatment underwent shotgun metagenomic analysis. Ultra-high-performance liquid chromatography-mass spectrometry was used to profile fecal bile acids. FMT-enriched bacteria that have been implicated in gut bile acid metabolism included *Desulfovibrio fairfieldensis* and *Clostridium hylemonae*. To identify candidate bacteria involved in gut microbial bile acid metabolism, we assessed correlations between bacterial species abundance and bile acid profile, with a focus on bile acid products of gut bacterial metabolism. *Bacteroides ovatus* and *Phocaeicola dorei* were positively correlated with unconjugated bile acids. *Bifidobacterium adolescentis*, *Collinsella aerofaciens*, and *Faecalibacterium prausnitzii* were positively correlated with secondary bile acids. Together, these data identify several candidate bacteria that may contribute to the metabolic benefits of FMT and gut bacterial bile acid metabolism that requires further functional validation.

## 1. Introduction

Type 2 diabetes mellitus (T2DM) continues to be a worldwide clinical challenge. The gut microbiota plays an important role in determining host metabolic health and has been associated with T2DM. Studies comparing the composition and function of the fecal microbiota from groups who had T2DM, impaired glucose tolerance, or normal glucose tolerance have reported distinct bacterial compositions. For example, *Roseburia* and *Faecalibacterium prausnitzii* are differentially enriched in T2DM [1]. Additionally, decreases in Bacteroidetes and increases in Actinobacteria and Firmicutes are associated with obesity [2,3]. Studies in germ-free mice show that transplantation of the gut microbiota from metabolically healthy vs. metabolically impaired donors transfers these metabolic phenotypes, pointing to a causative role for the gut microbiota in the pathogenesis of metabolic disease [4,5]. Therefore, the gut microbiome is an attractive target for the treatment and prevention of T2DM.

Fecal microbiota transplantation (FMT) is a potential method to target the gut microbiome for T2DM treatment and prevention [6]. FMT has been shown to successfully treat microbiota-related dysfunction, with the treatment of *Clostridioides difficile* infection being the most notable example of its successful therapeutic use [7,8]. To test the potential utility of FMT for the treatment of metabolic disease, our group previously studied patients with obesity, without metabolic impairment, treated with FMT or placebo. FMT did not induce weight loss but did successfully colonize the gastrointestinal tract of recipients and slowed the development of glucose intolerance compared with placebo, as assessed by mixed meal tolerance testing [9]. The mechanisms for this microbially induced improvement in glucose tolerance are unknown. However, a key mechanism by which the gut microbiota influences host metabolic health is through the production of metabolites, such as short-chain fatty acids (SCFAs) and unconjugated and secondary bile acids. Although SCFA concentrations were not altered by FMT, FMT increased gut bacterial bile acid metabolism compared to placebo resulting in a change in the bile acid profile that mirrored that of the lean donor [10]. 

Bile acids are a class of bioactive metabolites that signal through bile acid receptors, such as FXR and TGR5, to improve metabolic health. Bile acids are primarily metabolized by the liver and the gut microbiota. Primary bile acids are produced in the liver from cholesterol and are conjugated with taurine or glycine prior to secretion into the gut lumen. Primary bile acids are converted into secondary bile acids, deoxycholic acid (DCA) and lithocholic acid (LCA), by gut bacteria. Bile acids vary in their affinity for bile acid receptors. Therefore, alterations in the bile acid profile can influence metabolic health by altering bile acid receptor signaling. In particular, DCA and LCA are the strongest ligands for TGR5 [11,12,13,14]. Indeed, studies investigating the impact of gut microbiota on metabolic disease often identify gut bacterial bile acid production as a key mechanistic mediator. For example, dietary fiber supplementation has been reported to enhance gut bacterial 6-α-hydroxylation to improve metabolic phenotypes in mice [15]. Furthermore, recent work reports that enhancing gut bacterial bile acid deconjugation through the use of genetically modified microbes improves metabolic parameters in mice [16]. 

A key pathway in gut bacterial bile acid metabolism is the conversion of conjugated primary bile acids to secondary bile acids through deconjugation followed by 7-α-dehydroxylation. Primary bile acids are first deconjugated by the enzyme bile salt hydrolase (BSH) [17,18]. BSH expression has been identified across all major bacterial divisions and archaeal species in the gut, and elevations in BSH activity improve metabolic outcomes [19]. Furthermore, BSH activity enhances bacterial survival [19]. Therefore, BSH may also be a key determinant of the efficacy by which probiotics and FMT are able to successfully colonize the host [20]. However, the regulation of BSH expression is poorly understood. Unconjugated bile acids are converted to secondary bile acids through 7-α-dehydroxylation, which is a multi-step process that is less widely dispersed throughout the gut microbiome relative to BSH [17,18]. Nevertheless, the gut bacterial species and genes responsible for 7-α-dehydroxylation are still incompletely defined.

Research suggests FMT increases gut microbial diversity and the abundance of beneficial bacteria. Indeed, we found that patients who received FMT show sustained shifts in gut microbiota profiles toward those of the donor, as determined by 16S rRNA gene sequencing. Additionally, bile acid profiles resembled that of the donor [10]. Importantly, this shift in bile acid profile also coincided with a slowing of glycemic impairment compared with placebo [9]. The results from this clinical pilot, which evaluated the effectiveness of FMT in obese metabolically healthy patients, provide an ideal study set to identify gut bacteria involved in gut bacterial bile acid metabolism. Therefore, we assessed the impact of FMT on gut bacterial composition by metagenomics to better understand the dynamic alterations induced by FMT. Furthermore, we assessed the correlation between bacterial abundance determined by metagenomics with bile acid levels, assessed in the same samples, to identify putative bacterial species that may contribute to gut microbial bile acid metabolism.

## 2. Materials and Methods

### 2.1. Sample Collection

Secondary analysis was conducted on a single-center, double-blind, randomized, placebo-controlled pilot trial of FMT in obese metabolically normal/healthy patients (body mass index (BMI), 35 kg/m^2^ or higher without diabetes, metabolic syndrome, or non-alcoholic fatty liver disease). Briefly, patients were randomized 1:1 to receive FMT (via an induction dose of 30 FMT capsules followed by two maintenance doses of 12 capsules at week 4 and week 8) or an identical placebo capsule. A single healthy lean (BMI 17.5 kg/m^2^) donor was used to generate FMT capsules. A total of 22 patients were enrolled, 11 in each arm, and primarily female [10]. Samples collected at baseline (prior to FMT intervention) and after 4 weeks of intervention were available from 8 placebo and 11 FMT patients. Two patients in the placebo group withdrew from the study, and one placebo sample was unavailable at the 4-week time point. We focused on the 4-week time point because this was the time point at which the most substantial FMT-induced change in the bile acid profile was detected [10].

### 2.2. Microbiota Analysis

Fecal samples were shipped frozen to the George Washington University Genomics Core for processing. Each sample underwent DNA and RNA extraction in parallel from 250 mg of fecal material using a ZymoBIOMICS DNA/RNA Miniprep Kit (Zymo Research Corporation, Irvine, CA, USA). The resulting DNA was quality controlled using a Thermo Fisher Qubit 3.0 High Sensitivity DNA kit (Life Technologies, Carlsbad, CA, USA) and standardized to 0.2 ng/µL for library construction. Sequencing libraries were prepared, along with a ZymoBIOMICS Microbial Community DNA standard, using a Nextera XT DNA Library Prep kit (Illumina, San Diego, CA, USA) following Illumina’s recommended guidelines. Libraries were quality controlled using a Thermo Fisher Qubit 3.0 High Sensitivity DNA kit and an Agilent 2100 Bioanalyzer High Sensitivity DNA kit (Agilent, Santa Clara, CA, USA) and were subsequently sequenced as paired-end, 2 × 150 bp, using a NextSeq 500 Mid-Output kit (Illumina, San Diego, CA, USA), with a 1% phi X sequencing control spike-in.

### 2.3. Data Analysis and Statistics

Metagenomic shotgun sequencing read quality was assessed using FastQC and MultiQC [21,22], and host reads were filtered using kneaddata with low quality (Phred scores <28) ends and reads trimmed for downstream analyses. Functional pathways of associated microbes were determined using omePath [23]. Functional associations between metabolites, clinical phenotypes, and microbes were assessed using Tweedievers [24]. Data are presented as the mean ± SEM, and statistical analyses were performed using GraphPad Prism 9.4.1. Data were analyzed by non-parametric Wilcoxon matched-pairs signed rank test. Multiple corrections of statistical tests were applied using the Benjamini and Hochberg false discovery rate (FDR), and differences were considered significant at *p* ≤ 0.05 unless otherwise noted. 

## 3. Results

### 3.1. Microbial Diversity

Patient fecal samples obtained at baseline (prior to FMT intervention) and after 4 weeks of intervention were assessed by shotgun metagenomics. The metagenomic sequencing resulted in an average of 4,015,023 reads per sample, with a minimum of 2,280,276 reads and a maximum of 5,818,393 reads. Of these, 0.031235% were, on average, the host reads, leaving a minimum of 2,279,728 quality microbial reads for microbiome characterization with an average of 4,013,907 quality microbial reads per host individual. 

### 3.2. Impact of FMT on Gut Microbial Composition at 4 Weeks after the Initiation of Intervention

**Figure 1:**
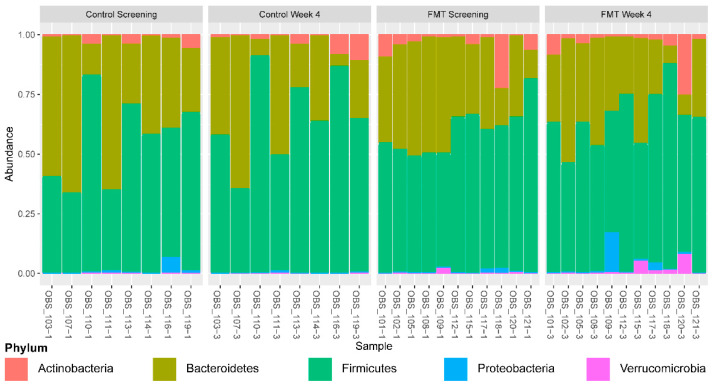
Phylum-level relative abundances across all individual patients. Patient identifiers ending in 1 represent baseline and ending in 3 represent the 4-week time point. Plots are faceted by treatment and time point.

**Figure 2:**
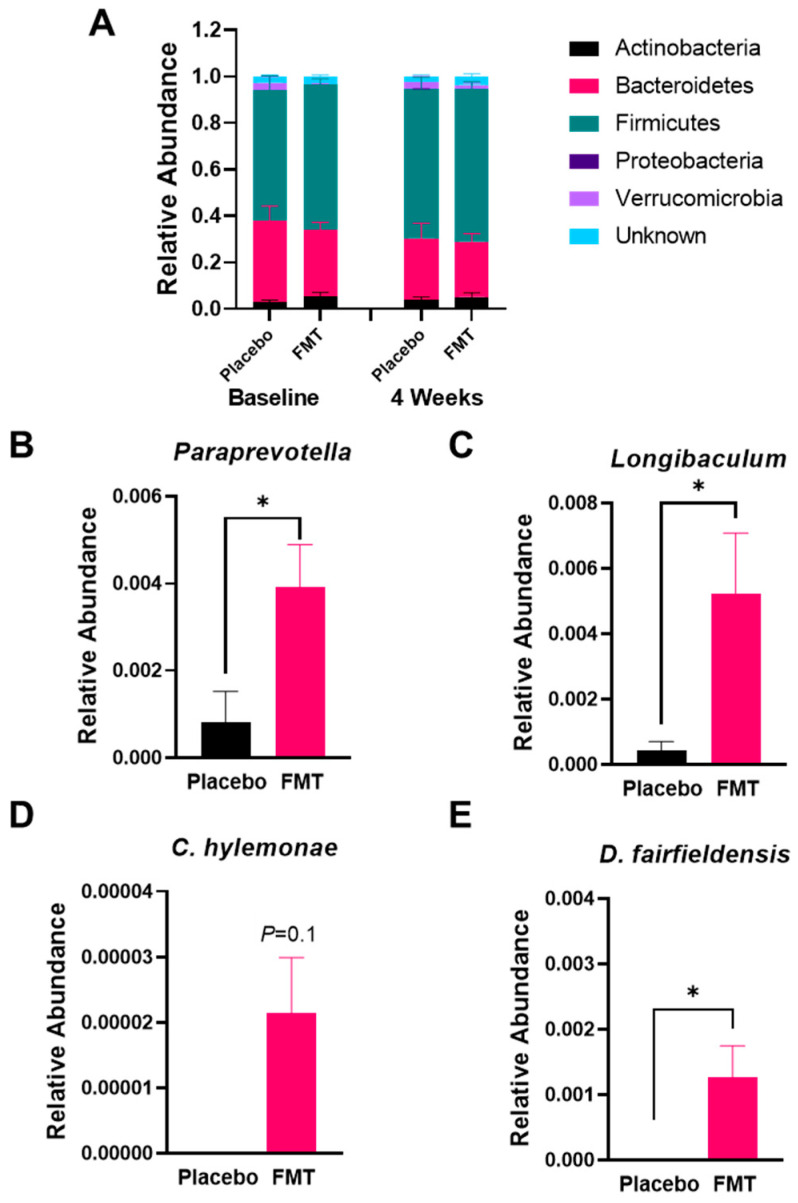
Impact of FMT on gut microbial composition. (**A**) Relative abundance of each bacterial phylum. Relative abundance of *Paraprevotella* (**B**) and *Longibaculum* (**C**), *Clostridium hylemonae* (**D**), and *Desulfovibrio fairfieldensis* (**E**) in fecal samples from placebo vs. FMT at baseline and after 4 weeks of treatment. Data presented as mean ± SEM. * *p* < 0.05.

There were no significant baseline clinical differences between FMT and placebo groups [10]. We focused on the fecal sample collected at baseline vs. 4 weeks after the initiation of FMT as this was the time point at which the changes in bile acid levels were most significant [10]. No significant differences in the relative abundance of bacteria were noted at the phyla level (Figure 1 and Figure 2A). At the genus level, FMT-enriched *Paraprevotella* and *Longibaculum* (Figure 2B,C, *p* < 0.05) compared to placebo. On the species level, FMT tended to increase the relative abundance of *Clostridium hylemonae*, a bacterial species known to convert primary to secondary bile acids (Figure 2D) [17,18]. Finally, FMT increased *Desulfovibrio fairfieldensis* compared with placebo (Figure 2E, *p* < 0.05). Of note, *Paraprevotella*, *Longibaculum, Clostridium hylemonae,* and *Desulfovibrio fairfieldensis* did not differ between groups at the baseline.

### 3.3. Impact of FMT on Gene Enrichment and Correlations with Secondary Bile Acid Production

**Figure 3:**
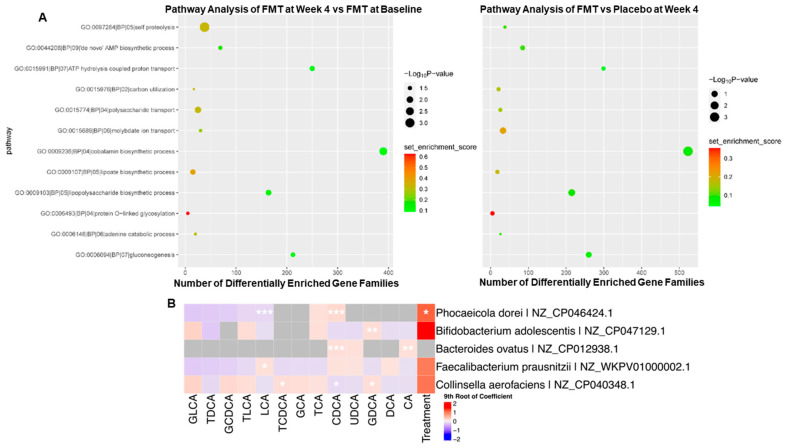
Association between bile acids and bacterial species. (**A**) Assessment of pathways enriched by FMT relative to baseline (**left**) and relative to placebo control after 4 weeks of treatment (**right**). (**B**) Bacterial species and strains that are correlated with gut bacterial-derived bile acids. * *p* < 0.2, ** *p* < 0.05, *** *p* < 0.001.

As some of the bacterial species enriched by FMT are implicated in bile acid metabolism, we next determined the effect of FMT on bacterial bile acid metabolic gene copy number. Starting with a broad overview of the impact of FMT on gut microbial gene abundance, we performed pathway analysis using *omePath* of the metagenomic data (Figure 3A). FMT-enriched genes involved in cell proteolysis pathways. Taking a closer look at bile acid metabolism, FMT did not impact gene abundance for most known gut bacterial bile acid metabolic genes, except for a reduction in *BaiB* and *BaiE*. These changes were noted at 4 weeks and not at baseline in the FMT group compared to the placebo. FMT did not impact the gene abundance of other genes involved in bile acid 7-α-dehydroxylation, including *BaiCD*, *BaiA2*, *BaiF*, and *BaiH*. Further work using metatranscriptomics is warranted to determine the impact of FMT on bacterial bile acid metabolic gene expression. 

To identify candidate bacteria involved in gut bacterial bile acid metabolism, we assessed correlations between bacterial species abundance and bile acid profile, with a focus on bile acid products of gut bacterial metabolism, namely unconjugated bile acids, and the secondary bile acids, DCA and LCA. Bile acid levels were measured in the same samples used for metagenomics analysis, as previously described [10]. The impact of FMT on bile acid levels in this sample set has been previously reported [10]. We focused on bacterial species that were positively correlated with bile acid sub-types that are produced, at least in part, through interactions with the gut microbiota with a *p*-value less than or equal to 0.08. Bacterial species that met these criteria are presented in Figure 3B. *Phocaeicola dorei* and *Bacteroides ovatus* were positively correlated with unconjugated chenodeoxycholic acid (CDCA) (*p* = 7.47 × 10^−45^ and 2.50 × 10^−8^). *Bifidobacterium adolescentis* and *Collinsella aerofaciens* were positively correlated with the production of DCA (specifically, the glycine-conjugated form, GDCA) (*p* = 0.0123 and 0.0634). The same strain of *B. ovatus* was positively correlated with unconjugated cholic acid (CA) (*p* = 0.0317). Lastly, *Faecalibacterium prausnitzii* was positively correlated with LCA (*p* = 0.0634). These data point to a potential role for *Phocaeicola dorei* and *B. ovatus* in bile acid deconjugation. Further, these data suggest that *Bif. adolescentis*, *C. aerofaciens,* and *F. prausnitzii* may play a role in the conversion of primary to secondary bile acids, which requires further functional validation. 

## 4. Discussion

In the present study, we utilized a fecal sample set from patients receiving FMT or placebo that exhibited alterations in gut bacterial bile acid metabolism to improve our understanding of the gut bacterial species involved in gut bacterial bile acid metabolism and how these pathways are dynamically regulated by FMT. Using metagenomics, we identified an enrichment of *Paraprevotella*, *Longibaculum*, *Desulfovibrio fairfieldensis*, and *Clostridium hylemonae* in response to FMT. Furthermore, through the assessment of correlations between fecal bile acid levels and bacterial species relative abundances, we identified *Bifidobacterium adolescentis*, *Bacteroides ovatus*, *Faecalibacterium prausnitzi*, and *Phocaeicola dorei* as potentially contributing to gut bacterial bile acid metabolism. Further work is needed to better understand secondary bile acid metabolism, its roles in metabolic disease, and how it can be manipulated through FMT. 

The effect of FMT to enrich *Paraprevotella, Longibaculum*, *C. hylemonae*, and *D. fairfieldensis* may have contributed to the effect of FMT to enhance gut microbial bile acid metabolism and/or slow the development of glucose intolerance. For example, members of the genera *Clostridium* are the predominant human intestinal species thought to perform 7-α-dehydroxylation of primary bile acids [25]. Additionally, *C. hylemonae* has been shown to convert CA into DCA in vitro [26]. Furthermore, *Paraprevotella* abundance was significantly increased after FMT in individuals with functional constipation whose changes in fecal microbiome compositions were measured before and after FMT. This increase in *Paraprevotella* abundance correlated with improved relief of clinical symptoms measured by three different clinical scales for constipation, suggesting *Paraprevotella* could improve metabolic dysregulation through gastric motility [27]. The role of *Longibaculum* in host metabolic health is poorly defined; however, dietary fiber supplementation has been shown to enrich for *Longibaculum* [28]. *D. fairfieldensis* is a Gram-negative anaerobic bacillus that has been implicated in bile acid metabolism. Interestingly, *D. fairfieldensis* bacteremia was found to be associated with choledocholithiasis in a case report [29]. Furthermore, a recent study reports that *Desulfovibrionales* are enriched in patients with cholelithiasis. Further, the administration of *Desulfovibrionales* to mice with antibiotic-induced depletion of the gut microbiome increased secondary bile acid production [30]. *Desulfovibrionales* can reduce taurine into H_2_S, which has been suggested to facilitate 7-α-dehydroxylation [31]. Together, these data suggest that *Desulfovibrionales*, and in particular *D. fairfieldensis*, may play a role in gut bile acid metabolism. Further, these data highlight the potentially important cooperative interactions among bacteria that facilitate gut microbial bile acid metabolism. 

In this study, we identified five bacteria that were positively correlated with gut microbiome-derived bile acids. Specifically, *Bifidobacterium adolescentis* and *Collinsella aerofaciens* were positively correlated with DCA. *Bacteroides ovatus* was positively correlated with unconjugated CDCA and CA. *Phocaeicola dorei* was positively correlated with unconjugated CDCA, and *Faecalibacterium prausnitzii* was positively correlated with LCA. Thus, our data suggest that *Phocaeicola dorei* and *Bacteroides ovatus* may perform bile acid deconjugation. Consistent with this, previous work reports that several *Bacteroides* strains, including strains of *B. ovatus*, express BSH [32]. Whether *Phocaeicola dorei* can perform bile acid deconjugation is unknown and requires further testing. Interestingly, a previous study identified a correlation between *Phocaeicola dorei*, also named *Bacteroides dorei*, and the risk of developing type 1 diabetes [33], suggesting a potential metabolic role for this species. The bacteria that were positively correlated with secondary bile acids (*Bifidobacterium adolescentis* and *Faecalibacterium prausnitzii*) are known to have BSH functions [34,35]. *Faecalibacterium prausnitzii* has been connected to anti-inflammatory effects and improvement of intestinal barrier function [36,37]. A role for *Collinsella aerofaciens* in the production of DCA has not been previously tested. However, *Collinsella aerofaciens,* previously known as *Eubacterium aerofaciens*, was found to have NADP-dependent 7-β-hydroxysteriod dehydrogenase activity, which is necessary for the production of hydrophilic secondary bile acids such as ursodeoxycholic acid [38].

The advantages of this study include the application of metagenomics to the analysis of the gut microbiome of individuals receiving FMT or placebo control. Additionally, the results from this secondary analysis are from individuals with obesity such that bacteria identified from this specific population can better inform FMT for the treatment of obesity and metabolic disease. Limitations of this study include the small sample size. While bile acid gene abundance was studied, metatranscriptomics analysis is needed to assess the impact of FMT on gene expression. Further work is needed to functionally validate bacteria identified as potentially contributing to the effects of FMT on gut bacterial bile acid metabolism. Together, these data demonstrate that FMT can alter the composition of bile acids and bacterial communities in the gut microbiome. 

## Data Availability

Sequence data generated by this project are deposited in NCBI Sequence Read Archive (SRA) and associated with BioProject PRJNA904790.
